# Prostaglandin F_2α_ FP receptor antagonist improves outcomes after experimental traumatic brain injury

**DOI:** 10.1186/1742-2094-10-132

**Published:** 2013-10-30

**Authors:** Alexander V Glushakov, Sean W Robbins, Connor L Bracy, Shuh Narumiya, Sylvain Doré

**Affiliations:** 1 Department of Anesthesiology, University of Florida College of Medicine, PO Box 100159, Gainesville, FL 32610, USA; 2 Department of Pharmacology, Kyoto University Faculty of Medicine, Yoshida, Sakyo-ku, Kyoto 606, Japan; 3 Departments of Neuroscience, Neurology, Psychiatry, and Center for Translational Research in Neurodegenerative Disease, University of Florida College of Medicine, 1275 Center Drive, Biomed Sci J493, PO Box 100159, Gainesville, FL 32610, USA

**Keywords:** AL-8810, Controlled cortical impact, Glial fibrillary astrocytic protein, G-protein-coupled receptors, Knockout mice, Prostaglandin F2a receptor, Traumatic brain injury

## Abstract

**Background:**

Injuries to the brain promote upregulation of prostaglandins, notably the proinflammatory PGF_2α_, and overactivation of their cognate G-protein-coupled FP receptor, which could exacerbate neuronal damage. Our study is focused on investigation of the FP receptor as a target for novel neuroprotective drugs in a preclinical animal traumatic brain injury (TBI) model.

**Methods:**

Accordingly, the effects of acute intraperitoneal post-treatment with selective FP antagonist AL-8810 were studied in wildtype (WT) and FP receptor knockout (FP^-/-^) mice after controlled cortical impact (CCI). Neurological impairments were evaluated using neurological deficit scores (NDS) and the grip strength test. Cortical lesions and overall brain pathology were assessed using immunohistochemistry.

**Results:**

Morphological analyses of cerebral vasculature and anastomoses revealed no differences between WT and FP^-/-^ mice. CCI produced cortical lesions characterized by cavitation, neuronal loss, and hematoma with a volume of 20.0 ± 1.0 mm^3^ and significant hippocampal swelling (146.5 ± 7.4% of contralateral) compared with sham (*P* < 0.05). Post-treatment with AL-8810 (1 to 10 mg/kg) had no significant effect on cortical lesions, which suggests the irreversible effect of primary CCI injury, but significantly reduced hippocampal swelling to a size not significantly different from the sham group. Post-treatment with AL-8810 at a dose of 10 mg/kg significantly improved NDS at 24 and 48 hours after CCI (*P* < 0.001 and *P* < 0.01, respectively). In the AL-8810 group, CCI-induced decrease in grip strength was three-fold (2.93 ± 1.71) less and significantly different than in the saline-treated group. The FP^-/-^ mice had significantly less hippocampal swelling, but not NDS, compared with WT mice. In addition, immunohistochemistry showed that pharmacologic blockade and genetic deletion of FP receptor led to attenuation of CCI-induced gliosis and microglial activation in selected brain regions.

**Conclusion:**

This study provides, for the first time, demonstration of the unique role of the FP receptor as a potential target for disease-modifying CNS drugs for treatment of acute traumatic injury.

## Background

Traumatic brain injury (TBI) is among the major causes of death and disability in the United States and current treatment is limited to supportive care. TBI is a complex disorder including four main pathological sequelae: contusions, diffuse axonal injury, hematoma, and subarachnoid hemorrhage causing secondary biochemical and metabolic changes that contribute to neuronal death [1]. Secondary brain injury involving excitotoxicity, oxidative stress, and neuroinflammation and edema plays a key role in the neurological outcome of TBI survivors [2]. Although the secondary injury following TBI is potentially treatable, there is no effective treatment currently available, and the discovery of new targets for TBI therapeutics remains critical. A few anti-inflammatory pathways are currently considered as a promising approach in TBI [3]. Furthermore, most of the therapeutic agents currently considered for TBI translation exhibit direct or indirect anti-inflammatory actions [4]. A growing body of experimental and clinical evidence suggests that the inducible cyclooxygenase-2 (COX-2) and prostaglandin synthetase enzymes play an important role in the neuroinflammatory cascades associated with neurotoxicity and neuronal damage during brain insults [5-8]. Increased COX-2 immunoreactivity has been demonstrated in microglia and neurons of the ischemic neonatal and adult human brain [9-11]. In addition, COX-2 upregulation has been reported in the cortex and hippocampus after experimental TBI, and this upregulation was associated with neuronal death [12-16]. Experimental evidence also has demonstrated that brain trauma causes an increased release of arachidonic acid, a COX-2 substrate involved in prostaglandin production [17,18]. Although COX-2 inhibition could be beneficial in TBI [12,14-16,19], clinical application of COX-2 inhibitors might be limited because of cardiovascular side effects [20]. Further studies on the downstream effectors of COX-2, such as prostaglandins and their respective receptors, have been previously been conducted and suggested that the certain prostaglandin receptor antagonists could be used as an alternative to COX-2 inhibitors [8]. Activation of several subtypes of prostaglandin receptors leads to increased intracellular calcium and neurotoxicity. The effects of PGF_2α_ are mediated via activation of its specific G-protein-coupled receptor, the FP receptor, and would result in neuronal intracellular calcium overload [21]. The roles of COX-2 and some prostaglandins are understood in stroke models [7,8,21,22]; however, the effect of PGF_2α_ and its FP receptor are not understood in TBI.

With the known physiological role of the FP receptor in calcium signaling [6,21], we hypothesized that changes in levels of PGF_2α_, due to increased COX-2 activity, and subsequent FP receptor overactivation may contribute to excitotoxic, hypoxic, and hemorrhagic damage [7]. Hence, our study was focused on the investigation of the FP receptor as a target for TBI drugs using its selective antagonist and FP receptor knockout (FP^-/-^) mice in a controlled cortical impact (CCI) model. Based on our preliminary data indicating that FP^-/-^ mice have significantly reduced infarct volume following stroke [21,22], we have tested whether genetic deletion of the FP receptor or its pharmacologic blockade with the selective antagonist AL-8810 [23] would limit brain damage and neurological outcome following brain trauma.

## Methods

### Animals

All animal protocols were approved by the University of Florida Animal Care and Use Committee. A total of 83 male wildtype (WT) and 20 male FP receptor knockout (FP^-/-^) C57BL/6 mice aged 2.0 ± 1.1 and 4.2 ± 1.5 months, respectively, was used. The mean body weight of WT and FP^-/-^ mice used for CCI was 23.3 ± 1.5 (n = 75) and 21.8 ± 3.9 g (n = 17), respectively.

### CCI procedures

CCI in mice was produced using PCI3000 PinPoint Precision Cortical Impactor (Hatteras Instruments, Cary, NC, USA) and a stereotaxic apparatus (David Kopf Instruments, Tujunga, CA, USA). Prior to all procedures, anesthesia was induced with 4% isoflurane in a 25% oxygen-in-air mixture. During all surgical procedures, mice were maintained on 2% isoflurane anesthesia via nose cone, and body temperatures were monitored using a rectal probe and were maintained with a controlled heating pad (Fine Science Tools, Vancouver, BC, Canada). After the skull was exposed with a central skin incision and soft tissue was removed with a cotton tip, a circular craniotomy of approximately 4 mm in diameter was made in the middle of the right parietal bone, about 0.5 mm from sagittal, coronal, and lambdoid sutures, leaving the dura intact under visual control using an Olympus SZ61 dissecting microscope (Olympus Corporation, Tokyo, Japan). The CCI parameters were as follows: impact tip diameter 3 mm, velocity 3 m/s, compression time 100 ms, and a compression distance of 1 mm. These parameters allow to produce experimental TBI of mild-to-moderate severity based on anatomical and neurobehavioral outcomes [24]. Sham mice underwent craniotomy only. After surgical procedures, the incision was closed using Reflex 7 skin closure system (CellPoint Scientific, Inc., Gaithersburg, MD, USA), each mouse received an intraperitoneal injection of warm saline to prevent dehydration, and the mice were transferred to a temperature-controlled recovery chamber for at least one hour.

### Drug treatments

In single regiment groups, AL-8810 (Cayman Chemical Co., Ann Arbor, MI, USA) was injected intraperitoneally after completion of all surgical procedures at two doses: 1 and 10 mg/kg. The animals with repeated treatment received three injections of AL-8810 at 10 mg/kg each. AL-8810 injections were done directly after surgery and at 24 and 48 hours. To perform the second and third injections, mice were briefly anesthetized with isoflurane to avoid potential injury to the surgery site due to handling. AL-8810 was dissolved in dimethyl sulfoxide at a concentration of 25 mg/mL, aliquoted, and stored at -20°C. Solutions for AL-8810 injection 25 or 250 μg/mL for AL-8810 doses of 1 and 10 mg/kg, respectively, were prepared in saline immediately before use.

### Neurological deficit scores (NDS)

Neurological function was assessed using a comprehensive 24-point NDS, slightly modified from Clark and colleagues [25]. The assessment included six individual tests, and each test was scored from 0 for normal performance up to 4 points with increasing severity, as summarized in Table 1. The sum of scores from the individual tests was reported as the NDS. All scoring was performed at the time of test and was verified offline using video recordings. The NDS was assessed at least at 24 hours after termination of anesthesia in single and repeated drug- or saline-treatment groups. No detectable differences were observed between saline-treated sham groups with single and repeated treatments, indicating that brief anesthesia had no effect on NDS; thus, the NDS data from saline-treated treated mice that underwent CCI were pooled together.

**Table 1:** Neurological deficit scoring

**Test**	**Body symmetry**	**Gait**	**Circling behavior**	**Climbing**	**Front limb symmetry**	**Compulsory circling**
Score	Mouse allowed to move freely on the elevated open rectangular plain surface for two to five minutes			Mouse placed on the gripping surface with 45° angle for 60 seconds	Mouse suspended by tail for 30 seconds	Mouse suspended by tail with front limbs on bench for 30 seconds
0	normal	normal	not present	normal	not present	not present
1	tilting on one side	stiff, inflexible	predominant one-sided turns	climbs with strain	slight asymmetry	tendency to turn to one side
2	moderate asymmetry	limping	circles to one side not constantly	holds on slope	marked asymmetry	circles to one side
3	prominent asymmetry	trembling	circles to one side constantly	slides down slope, unsuccessful effort to prevent fall	prominent asymmetry	pivots to one side sluggishly
4	extreme asymmetry	does not walk	pivoting, swaying, or no movement	slides down immediately, no effort to prevent fall	slight asymmetry, no body or limb movement	does not advance

### Grip strength test

Forelimb strength measurements in mice were carried out using the Animal Grip Strength System (San Diego Instruments, San Diego, CA, USA). The mouse was placed over the grid by the tail so that its forepaws were allowed to grasp the steel bar and it was then pulled backward until the grip was released. Each test consisted of three consecutive trials. Between trials, each mouse was allowed to rest for one minute. The data were reported as the average value of maximal force recorded before the mouse released the bar. Baseline grip strength values were obtained in each animal one day before CCI or sham procedure.

### Histological procedures and analyses

Mice were euthanized and transcardially perfused with 4% paraformaldehyde in PBS. Brains were removed, post-fixed with the perfusion solution, and incubated for at least 24 hours in 30% sucrose/PBS solution. A series of eight 30-μm thick coronal sections were obtained throughout the entire brain and processed for histological analysis. To quantitate brain pathology, cresyl violet staining was used. Cortical infarct volume was quantified using a slightly modified procedure described elsewhere [26]. Briefly, cortical lesion volume was calculated from injured areas located and measured in eight brain sections spaced within 0.5 mm apart from the same animal. The lesions characterized by cavitation, neuronal loss, and hematoma. These cresyl violet-stained slides were also used for assessment of hippocampal edema. Hippocampal swelling was quantified using three brain sections from the same slides between 1 and 2 mm posterior to the bregma, representing injured brain regions within midline of focal impact. The areas of hippocampi in each hemisphere were measured and presented as the mean ratio between values of ipsilateral to contralateral sides. Immunohistochemistry for ionized calcium-binding adapter protein 1 (Iba1) and glial fibrillary acidic protein (GFAP) immunohistochemistry were performed using polyclonal rabbit anti-Iba1 (1:1,000 dilution; Wako Bioproducts, Richmond, VA, USA) and anti-GFAP (1:2,000 dilution; DAKO, Carpinteria, CA, USA) primary antibodies, respectively, avidin-peroxidase-labeled biotin complex secondary antibodies (1:1,000 dilution; BA-500, Vector Laboratories, Burlingame, CA, USA), and Vectastain ABC and DAB SK-4100 kits (Vector Laboratories, Burlingame, CA, USA) according to the manufacturer’s instructions. All slides were scanned using ScanScope CS (Aperio Technologies, Inc., Vista, CA, USA) and analyzed using ImageScope software (Aperio Technologies, Inc., Vista, CA, USA).

### Visualization of vasculature and analysis of anastomosis

Mice were deeply anesthetized with a lethal dose of isoflurane and were perfused via the left ventricle with 5 mL of cold PBS, followed by 1 mL of black latex paint with flow rates not to exceed 1 mL/min. The brains were post-fixed in 10% paraformaldehyde for 24 hours at 4°C prior to imaging of the dorsal cortical surface. Images of the dorsal surface were obtained using a desktop endoscope/microscope. Anastomosis of the cortical surface was determined by tracing the distal branches of the anterior cerebral artery (ACA) and middle cerebral artery (MCA) to the point of anastomosis. The point of anastomosis was defined as the narrowest point along the vessel connecting the ACA and MCA, or halfway between the nearest branch points [27]. Each anastomosis was marked with a dot. All anastomoses were connected with a line to form the line of anastomosis. Next, the distance from the midline was determined by measuring, from the midline to the line of anastomosis, every millimeter from the frontal pole to the end of the cerebral cortices [28].

### Statistical analyses

The statistical comparisons among multiple groups were done using one-way ANOVA followed by Tukey's multiple comparison test. Differences between two groups were determined by two-tailed paired or unpaired Student’s *t-*tests. Normality assumption testing was performed using the Kolmogorov-Smirnov test. If data were non-parametric by nature (for example, data were already ordered according to qualitative ranks such as NDS), the Kruskal-Wallis ANOVA on ranks sum test was used to compare differences between multiple groups and the Wilcoxon rank sum test was used to compare differences between two groups to compare measurements before and after an intervention (that is, single and repeated drug treatments) in the same animals. Data are presented as the mean ± standard error or mean ± standard deviation and *P <* 0.05 was considered as statistically significant [29].

## Results

### Effect of selective FP antagonist AL-8810 on the anatomical outcomes

To determine the effects of CCI in all experiments, the treated animals were compared with sham-operated animals that had undergone craniotomy only. To evaluate the FP receptor as a novel target, selective FP receptor antagonist AL-8810 was administered intraperitoneally within ten minutes after CCI, as we previously did in ischemic stroke models [21,22].

**Figure 1:**
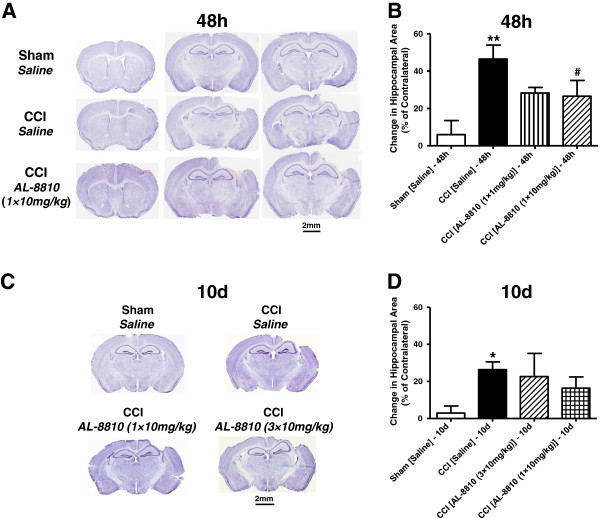
**Brain pathology after controlled cortical impact (CCI): the effect of FP receptor blockade. (A)** Representative photographs of cresyl violet-stained brain sections from sham and CCI-injured WT mice demonstrating cortical injury and hippocampal swelling in the areas between bregma one and two 48 hours after injury. **(B)** Quantitative analysis of the hippocampal area demonstrating a significant increase in relative hippocampal areas normalized to contralateral side in the ipsilateral hemisphere and significant decrease of hippocampal swelling in AL-8810- (10 mg/kg) treated mice 48 hours after CCI. **(C)** Representative photographs of cresyl-violet stained brain sections from sham and CCI-injured WT mice demonstrating cortical cavitation and remaining hippocampal swelling in the areas ten days after injury. **(D)** Quantitative analysis of relative hippocampal areas normalized to the contralateral side in saline- and AL-8810- (10 mg/kg) treated WT mice that underwent CCI or sham. The data demonstrate a significant hippocampal swelling ten days after injury compared with sham group, whereas there are no significant differences between relative hippocampal area values between two treatment groups that underwent CCI, between sham and either of these treatment groups, or between CCI groups with saline and drug treatments. Data are presented as mean ± SEM, *P < 0.05, **P < 0.01, versus saline-treated sham group, and ^#^*P* < 0.05, versus saline-treated CCI group, one-way ANOVA followed by Tukey's multiple comparison test (n = 7 to 9).

To determine if the FP receptor blockade will improve short-term anatomical outcome following CCI, brain sections were analyzed 48 hours after surgery. Mice were randomly assigned to four groups: sham, CCI saline control, and two AL-8810 treatment groups with doses of 1 and 10 mg/kg. At the 48-hour time point, CCI caused complex cortical lesions, including hematoma, decrease in cellular density in surrounding areas, and loss of cortical tissues referred to as cavitation. In saline-treated animals, CCI caused cortical injury with a relative volume of 20.0 ± 1.0 mm^3^, whereas no detectable cortical injury was observed in sham animals. Acute post-treatment with AL-8810 at both doses had no significant effect on cortical lesions, which suggests the irreversible effect of primary mechanical CCI injury. Also, AL-8810 did not cause any detectable changes in brain morphology in the sham animals (n = 3, Additional file 1: Figure S1A). In addition to cortical injury, significant hippocampal swelling (146.5 ± 7.4% of contralateral) was observed in all saline-treated CCI animals compared with sham (*P* < 0.05, n = 4). Post-treatment with AL-8810 at both doses reduced CCI-induced hippocampal swelling to levels not significantly different from the sham group (Figure 1, A and B). However, a significant difference between AL-8810- and saline-treated animals that underwent CCI was observed only at a dose of 10 mg/kg. To test whether the beneficial effects of a single post-treatment with AL-8810 (10 mg/kg) following CCI would be sustained for extended time periods, anatomical assessments were performed ten days after injury. To test whether repeated AL-8810 treatment would have additional benefits, this compound was administered at a dose of 10 mg/kg three times in a separate group. In this treatment group, the first AL-8810 injection was administered post-CCI, as in the single treatment group, and then two additional injections were given once a day during the next two days. At this late time point in the CCI group, the lesions were characterized by structurally defined cortical cavitation (Figure 1, C and D) and the significant hippocampal swelling was still present, though it was less prominent compared with the 48-hour time point. Ten days after injury, hippocampal swelling in the CCI group had a value of 126.39 ± 4.110 (n = 8) of the contralateral side and was significantly lower than the value at the 48-hour time point (*P* < 0.05, Student independent *t*-test between two CCI groups). Post-treatment with AL-8810 using both treatment regiments showed a tendency to decrease the CCI-induced hippocampal swelling to levels not significantly different from the sham group; however, there was no significant difference between single and repeated treatments or between saline and treatment groups of mice who underwent CCI.

### Effect of selective FP antagonist AL-8810 on the neurobehavioral outcomes

**Figure 2:**
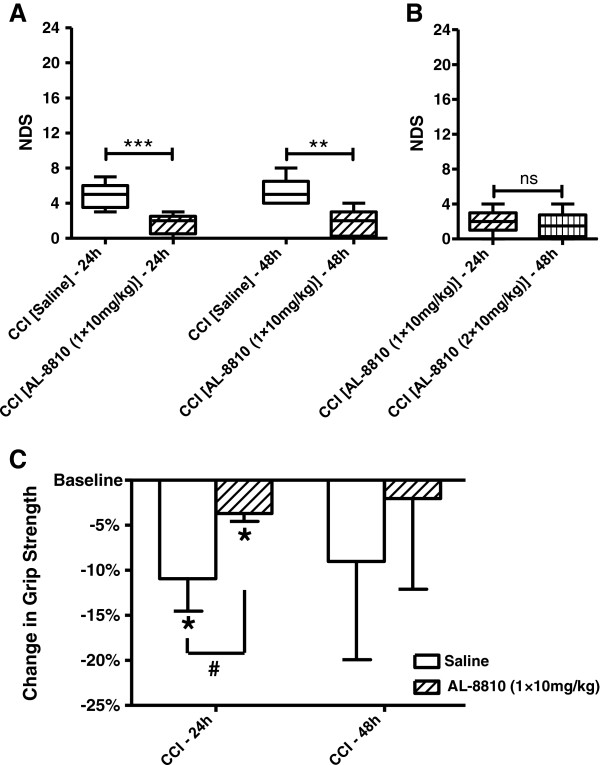
**FP receptor blockade improves behavioral outcomes after controlled cortical impact (CCI). (A)** Neurological deficit scores (NDS) in CCI-injured WT mice 24 and 48 hours after injury. Post-treatment AL-8810 (10 mg/kg) significantly improved NDS at both time points, **P < 0.01, ***P < 0.001, Kruskal-Wallis ANOVA on ranks followed by Dunn's multiple comparison test (n = 5 to 9). **(B)** Comparison of NDS between a single dose of AL-8810 (10 mg/kg) 24 hours after injury and two doses of AL-8810 (10 mg/kg, once a day) 24 and 48 hours after injury in the same mice that underwent CCI. The data were obtained in a separate cohort of mice. ‘ns’ denotes not statistically different, Wilcoxon matched-pairs signed rank test (*P* = 0.6, n = 8). **(C)** Changes in grip strength in CCI-injured WT mice 24 and 48 hours after injury. Post-treatment AL-8810 (10 mg/kg) significantly improved CCI-induced impairment in grip strength at 24 hours. *P < 0.05, paired Student’s *t*-test versus baseline. ^#^*P* < 0.05, one-way ANOVA followed by Tukey's multiple comparison test, n = 3 in each group.

Neurobehavioral assessment was performed at 24 and 48 hours after CCI or sham. CCI caused marked neurological impairment in saline-treated CCI mice as reflected in increased NDS, whereas no or only marginal neurological symptoms were observed in the sham animals. Figure 1A demonstrates a significant neurological impairment following CCI at the 24- and 48-hour time points compared with sham animals. The NDS in CCI animals was not significantly different between the two tested time points. Our data indicate that a single post-treatment with AL-8810 at a dose of 10 mg/kg significantly improved the NDS at both tested time points 24 and 48 hours after CCI, whereas the effect of a single 1 mg/kg dose was not significant (Figure 2A). To determine if repeated treatment with AL-8810 would have additional benefits compared with a single treatment, a separate group of mice that had NDS assessed at 24 hours and that had received one treatment immediately after CCI were given an additional treatment 24 hours after CCI and the first NDS assessment according to the repeated treatment protocol used in this study, and the NDS in these mice was assessed second time at 48 hours after CCI (Figure 2B). The data demonstrated that following a second AL-8810 treatment, NDS values were not further improved compared with a single treatment.

The grip test was used as an additional test to assess neuromuscular function following CCI by measuring maximal muscle strength of forelimbs as a primary phenotype screen. To measure forelimb strength, three sets containing three consecutive trials each were carried out before the CCI procedure and 24 and 48 hours after CCI (Figure 2C). The mice were randomly divided in two groups: control and experimental. The average baseline values were 186.11 ± 3.89 g and 193.67 ± 10.65 g in control and experimental groups, respectively, and these values were not significantly different (data presented as mean ± standard deviation, *P* = 0.3, independent Student’s *t*-test, n = 3 in each group). Both groups were subjected to CCI, and the experimental group received single intraperitoneal injections of AL-8810 at a dose of 10 mg/kg, whereas the control group received single injections of saline. CCI caused a significant decrease in the grip strength 24 hours post-CCI in the saline-treated group, but to a significantly lesser extent in the AL-8810-treated group compared with the corresponding baseline values in these groups. This experiment showed that in the AL-8810-treated animals 24 hours after CCI, the decrease in the grip strength was significantly improved to about 50% of the value observed in saline-treated animals, although at 48 hours, the changes in grip strength values were no longer significantly different.

### Effect of FP receptor knockout anatomical and neurobehavioral outcomes following CCI

Based on our published data demonstrating the neuroprotective effect of genetic FP receptor deletion in ischemic stroke models [21,22], we have tested this hypothesis to see if similar improvement could be achieved in the TBI model. Cortical lesion size and hippocampal swelling in FP^-/-^ mice were assessed 48 hours following CCI using cresyl violet staining on 30-μm cryostat brain sections, and behavioral outcomes were assessed using NDS.

**Figure 3:**
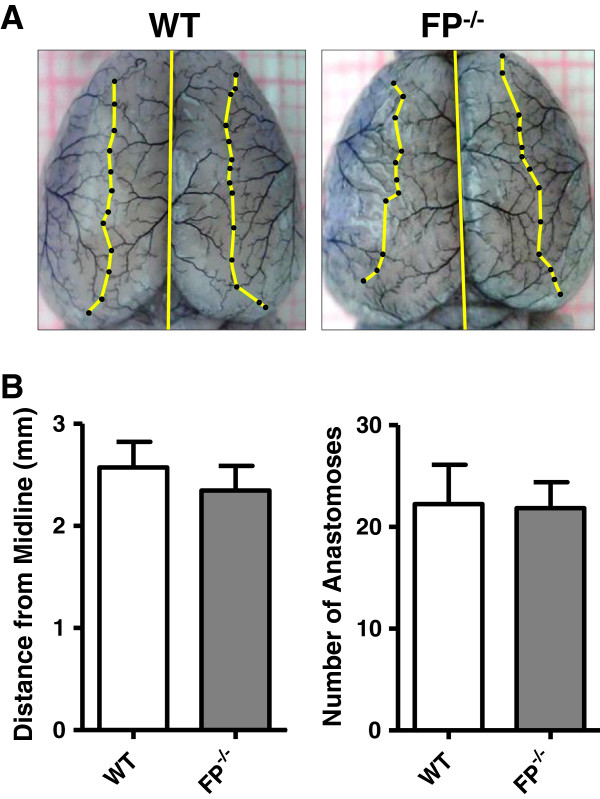
**Distance from the midline and number of anastomoses in wildtype (WT) and FP receptor knockout (FP**^**-/-**^**) mice. (A)** Representative photographs of cortical surface of brains from WT and FP^-/-^ mice perfused with black latex paint. Yellow lines and black dots illustrate lines and points of anastomosis. Bar graphs represent mean values of total number of anastomoses for both hemispheres **(B)** and mean distances from midline **(C)** in WT (n = 9) and FP^-/-^ (n = 6) mice. Data presented as mean ± standard deviation. No significant differences were observed between the values in WT and FP^-/-^ mice when using unpaired Student’s *t*-test.

Based on the data that strain-dependent variability in cerebral vascular anatomy might be associated with different susceptibility to cerebral ischemia [30], and knowing the importance of vascular damage in TBI, we looked at anatomical differences between WT and FP^-/-^ mice in the major cortical arteries that might potentially affect outcome in the CCI model. Detailed morphological analysis of ACA and MCA using black latex paint showed that there were no significant differences in total numbers of anastomoses and the location of the line of anastomosis between the two groups (Figure 3).

**Figure 4:**
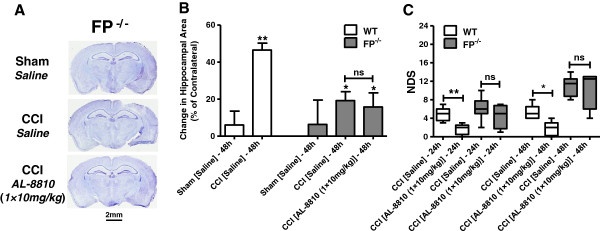
**The effects of FP receptor deletion on the anatomical and behavioral outcomes after controlled cortical injury (CCI). (A)** Representative photographs of cresyl violet-stained brain sections between 1 and 2 mm posterior from bregma from sham- and CCI-injured FP^-/-^ mice 48 hours after injury. **(B)** Quantitative analysis of related hippocampal areas normalized to contralateral side in sham, and saline- and AL-8810- (10 mg/kg) treated mice that underwent CCI. Data are presented as mean ± SEM, **P < 0.01, versus saline-treated WT sham group, and ^#^*P* < 0.05, versus saline-treated WT CCI group. ‘ns’ denotes not statistically different, one-way ANOVA followed by Tukey's multiple comparison test (n = 4 to 9). **(C)** Neurological deficit scores (NDS) in CCI-injured FP^-/-^ mice in comparison with WT mice 24 and 48 hours after injury. Post-treatment with AL-8810 (10 mg/kg) had no significant effect on NDS in CCI-injured saline or AL-8810-treated FP^-/-^ mice; there was also no significant difference between FP^-/-^ and WT mice who underwent CCI at both time points. *P < 0.05, **P < 0.01. ‘ns’ denotes not statistically different, Kruskal-Wallis ANOVA on ranks followed by Dunn's multiple comparison test (n = 4 to 6).

Hippocampal swelling in FP^-/-^ mice that underwent CCI were not significantly different from sham FP^-/-^ animals. However, hippocampal swelling was significantly lower compared with saline-treated WT mice from the CCI group. Post-treatment with 10 mg/kg AL-8810 also had no additional effect. In addition, a group of FP^-/-^ mice were post-treated with AL-8010 to test the specificity of the protective effect of this drug candidate (Figure 4, A and B). No significant differences in anatomical outcomes between AL-8010 and saline treatments were observed in the FP^-/-^ mice.

Also, when using severe CCI parameters, NDS 24 and 48 hours after injury was not significantly different between saline-treated WT and FP^-/-^ mice (Figure 4C). In the FP^-/-^ mice, post-treatment with AL-8810 at the highest dose of 10 mg/kg also had no significant effect on the NDS.

### Effect of selective FP antagonist AL-8810 on the microglial activation and astrogliosis following CCI

**Figure 5:**
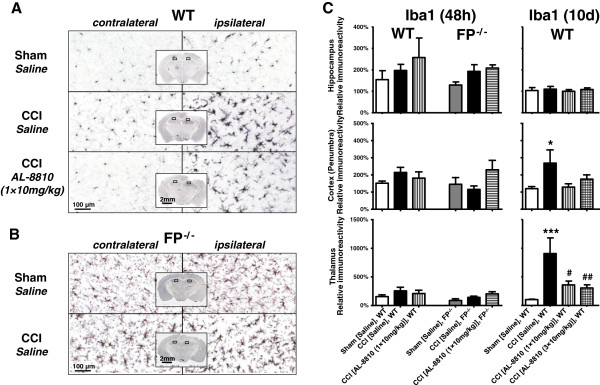
**Effects of genetic deletion and blockade of FP receptor on the ionized calcium-binding adapter protein 1 (Iba1) expression after controlled cortical injury (CCI). (A and B)** Representative photographs of Iba1 immunohistochemistry on hippocampal brain sections from sham- and CCI-injured WT (A) and FP^-/- ^**(B)** mice 48 hours after injury. Rectangular selections in the inserts denote magnified regions. **(C)** Bar graph represents quantitative analysis of Iba1 immunoreactivity in cortical penumbra, hippocampus, and thalamus in saline- and AL-8810- (10 mg/kg) treated WT and FP^-/-^ mice that underwent CCI or sham 48 hours and 10 days following injury. All data are normalized to the corresponding value of the contralateral side. Data are presented as mean ± SEM, *P < 0.05, **P < 0.01 versus saline-treated WT sham-operated animals, ^#^*P* < 0.05, ^##^*P* < 0.01, versus saline-treated WT CCI group, one-way ANOVA followed by Tukey's multiple comparison test (n = 3 to 7).

Knowing the important role of microglia and astrocytes in TBI [31], to investigate putative protective mechanisms of AL-8810 in the brain, we performed immunohistochemical studies to detect changes in the brain levels of microglial and astrocytic markers in WT mice 48 hours and 10 days following injury. In addition, to study the role of the FP receptor in the brain following brain injury, and possible involvement of this receptor in microglial activation and astrogliosis, the changes in the brain levels of microglial and astrocytic markers were studied in the FP^-/-^ mice 48 hours after CCI, similar to that of the WT mice. Forty-eight hours after injury, CCI caused apparent microglial activation detected as increased Iba1 immunoreactivity in the surrounding areas of cortical injury, referred to as a penumbra, as well as in the ipsilateral hippocampus and some thalamic regions within lateral dorsal and posterior nuclei compared to corresponding contralateral areas. In the CCI-injured animals, increased Iba1 immunoreactivity was accompanied by morphological changes in the immunopositive cells. Figure 5A shows microphotographs that represent typical morphological changes in the Iba1-positive cells in the hippocampus, suggesting activation of microglia in the ipsilateral compared with contralateral area. A slight increase was shown in Iba1 levels in the ipsilateral cortical area surrounding craniotomy, but not in other brain regions, and was also observed in sham animals. However, the changes in the Iba1 levels in sham animals were substantially lower than in the cortical penumbra of the animals from the CCI group. In the AL-8810-treated animals, the morphological changes in the microglia were less strongly pronounced than in the saline-treated group. However, no statistical differences between relative immunoreactivities, normalized to the corresponding areas in the contralateral side, were detected between these two groups at 48 hours (Figure 5C). No apparent changes in the Iba1 immunoreactivity were observed between contralateral sides of sham- and CCI-treated FP^-/-^ mice (Figure 5B). Ten days after injury, Iba1 immunoreactivity was significantly increased in the cortical penumbra in the saline-treated CCI animals but not in AL-8810 treated-animals, whereas in the hippocampus, Iba1 immunoreactivity was decreased to the control level at this time point. The most significant improvement with single and repeated AL-8810 treatment was observed in the susceptible thalamic regions (Figure 5C).

**Figure 6:**
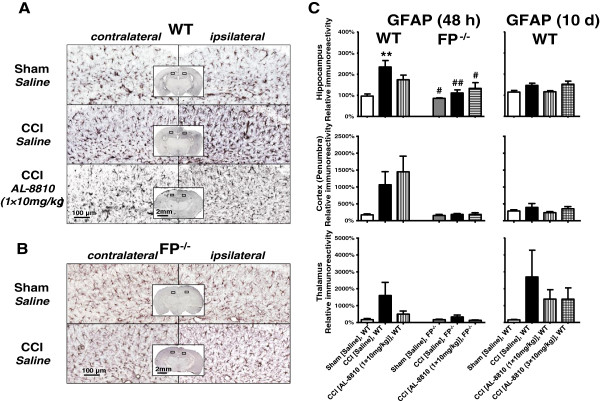
**Effects of genetic deletion and blockade of FP receptor on the glial fibrillary acidic protein (GFAP) expression after controlled cortical injury (CCI). (A and B)** Representative photographs of GFAP immunohistochemistry on hippocampal brain sections from sham- and CCI-injured WT (A) and FP^-/- ^**(B)** mice 48 hours after injury. Rectangular selections in the inserts denote magnified regions. **(C)** Bar graph represents quantitative analysis of GFAP immunoreactivity in cortical penumbra, hippocampus, and thalamus in saline- and AL-8810- (10 mg/kg) treated WT and FP^-/-^ mice that underwent CCI or sham 48 hours and 10 days following injury. All data are normalized to the corresponding value of the contralateral side. Data are presented as mean ± SEM, ***P < 0.001 versus saline-treated WT sham group, ^#^*P* < 0.05, ^##^*P* < 0.01, versus saline-treated WT CCI group, one-way ANOVA followed by Tukey's multiple comparison test (n = 3 to 7).

To study reactive gliosis, we used GFAP immunoreactivity. Similar to a microglial marker, the GFAP levels were considerably increased in the cortical penumbra, as well as in the ipsilateral hippocampus and thalamic areas compared with the corresponding contralateral areas in the CCI-treated animals at both time points used in the study. A small increase in the GFAP was observed in the cortical area surrounding craniotomy in the sham WT animals, and similar increases were observed in the sham- and CCI-treated FP^-/-^ mice (Figure 6A). No detectable differences in the GFAP immunoreactivities between ipsilateral and contralateral hippocampus and thalamic regions were observed in sham-operated WT animals or in either sham- or CCI-injured FP^-/-^ mice. In the cortical penumbra and hippocampus, the increase in the related GFAP immunoreactivities was observed at 48 hours, but not 10 days, after injury, whereas in the thalamus, the increased immunoreactivity remained up to 10 days. The changes in GFAP levels in the FP^-/-^ mice were substantially lower compared with the CCI-treated WT animals (Figure 6B). In FP^-/-^ mice, no significant changes in GFAP immunoreactivity were detected in AL-8810 treatment groups compared with the saline group following CCI (Figure 6C).

## Discussion

This study provides, for the first time, clarification of the respective role of the calcium-modulating FP receptor as a potential target for disease-modifying CNS drugs in the treatment of acute brain injury. Our data demonstrated significant anatomical and neurological improvements following pharmacological blockade of the FP receptor with a small-molecule-selective antagonist in a preclinical CCI mouse model, suggesting the involvement of this receptor in neuropathological consequences of acute brain trauma. In WT mice, post-treatment with AL-8810 following CCI significantly improved NDS and ameliorated grip strength impairment following CCI, and decreased CCI-induced hippocampal swelling and the level of markers of gliosis and microglial activation (that is, GFAP and Iba1) in susceptible brain regions 48 hours and 10 days following experimental brain injury. Furthermore, morphological analyses of cerebral vasculature and anastomoses in WT and FP^-/-^ mice confirmed that there are no differences between these strains. In the FP^-/-^ mice, the hippocampal swelling and accumulation of astrocytic and microglial markers in the brain were substantially lower than in WT mice, and post-treatment with AL-8810 had no additional benefits in FP^-/-^ mice, indicating the selectivity of pharmacological action of this drug candidate on the FP receptor. Importantly, the beneficial effects of FP antagonist were achieved with a single systemic treatment, suggesting translational potential for this compound in clinical use, particularly in critical care settings for the management of TBI.

Our previous data suggested a potential therapeutic role for PGF_2α_ FP receptor blockade in preclinical ischemic stroke models [21,22]. Despite different epidemiology and etiologies of stroke and TBI, both conditions share many common pathophysiological features, including formation of ischemic penumbra and brain edema [32]. TBI is a complex disorder that causes brain damage through several coexisting mechanisms, including primary and secondary excitotoxicity, ischemia, brain hemorrhage, and after toxicity that has been caused by hemoglobin breakdown products, diffuse edema, and upregulation of proinflammatory mediators [33]. Vascular damage plays an important role in TBI and is a primary cause of hemorrhage in different brain regions to the extent correlated with TBI severity [34], extensive glutamate release [35], and cortical necrosis [34]. The hemorrhagic component itself could trigger secondary biochemical cascades, exacerbating primary brain damage involving oxygen free radicals, membrane lipid peroxidation, glutamate receptor upregulation, and excitotoxicity [36,37]. Neuroinflammatory pathways involving COX-2 upregulation have been considered to be involved with pathological sequelae of TBI [12,13]. Experimental reports and clinical data suggest that COX-2 upregulation and subsequent increases in the levels of several classes of prostaglandins, notably PGF_2α_, are involved in numerous neurological disorders. In mammalians, expression of the FP receptor has been demonstrated in whole brain homogenates [38], brain synaptosomes [39], and cerebral microvessels [40]. In addition, it has been reported that the FP receptor is expressed in cultured cortical neurons [41] and astrocytes [42].

The role of the FP receptor in the brain is not well understood. It has been shown that this receptor is involved differently in the pathological pathways in animal models of stroke [21,22] and seizures [43]. In addition, PGF_2α_ induces cerebral vasoconstriction in adults and newborns, though in the latter case to a substantially lesser extent [44,45]. A significant increase in PGF_2α_ was demonstrated in the cerebrospinal fluid (CSF) of patients with stroke and subarachnoid hemorrhage when samples were collected shortly after the cerebral attack, which then decreased with the regression of clinical symptoms [46,47]. However, other studies found no relationship between PGF_2α_ level in lumbar CSF and neurological deficit [48]. Experimental study further suggests that the PGF_2α_ concentration in lumbar CSF might not reflect its intracranial level depending on the size and location of the hemorrhage [47]. Clinical data indicate the COX-2 upregulation in hippocampal biopsies from patients with therapy-refractive temporal lobe epilepsy [49], which is consistent with the previous findings of increased concentrations of PGF_2α_ in the CSF of epilepsy patients [46,50]. Increased CSF PGF_2α_ levels have also been observed in infants and children with febrile convulsions [51], which have been recently proposed as a risk factor for development of temporal lobe epilepsy [52,53]. Other evidence supporting the proconvulsive properties of PGF_2α_ include several cases of seizures and abnormal electroencephalographic changes associated with the clinical use of PGF_2α_ analogs to terminate pregnancy that have been reported in clinical practice [54-57]. Nevertheless, action PGF_2α_ is disputed and the FP receptor role still remains controversial [58]. Several contrary experimental reports demonstrated that administration of PGF_2α_ caused seizure aggravation [59] and abolished the anticonvulsant effects of other prostaglandins [60,61], whereas others suggested that elevated levels of PGF_2α_ might have anticonvulsant action [43,62]. The level of arachidonic acid, a precursor of prostaglandin synthesis, significantly increases in the cortex and hippocampus after CCI in rodents, and it has been suggested that arachidonic acid metabolites, including PGF_2α_, may play a role as mediators in the blood–brain barrier breakdown [63,64], which might be associated with edema formation in TBI [65]. On the molecular level, activation of the FP receptor initiates several events, including stimulation of the phospholipase C/IP_3_R/Ca^2+^ signaling pathway [6,21]. By this means, supraphysiological levels of PGF_2α_ may potentially lead to intracellular calcium overload [21], which could consequently lead to excessive release of excitatory neurotransmitters, or could even trigger neuronal necrosis via activation of calpains [66].

In this study, we used male WT and FP^-/-^ mice of the same C57BL/6 background. Strain-dependent differences in the response to the experimental brain trauma have been reported in rats [67,68] and mice [69,70]. Some studies in mouse stroke models have demonstrated that strain-dependent differences in cerebrovascular anatomy are associated with susceptibility to cerebral ischemia [30], although others suggest an involvement of intrinsic genetic determinants in ischemia and glutamate excitotoxicity-induced cell death [28,71]. Our previous data, obtained in an ischemia model, indicated that genetic deletion of the FP receptor does not affect the vital physiological parameters in mice. No substantial differences in cerebral blood flow, body temperature, mean arterial blood pressure, blood gases (PaO_2_, PaCO_2_), or pH between WT and FP^-/-^ mice were observed before, during stroke, or after reperfusion [22]. This study also demonstrated no significant differences in anastomoses of two major cerebral arteries. Taken together, these data suggest that differences between anatomical and neurological outcomes between WT and FP^-/-^ mice are associated with the FP receptor deletion.

In this study, AL-8810 significantly improved anatomical and neurological outcomes in a preclinical TBI model. It is well known that regional vulnerability of the brain is characteristic for TBI. In unilateral TBI models performed in adult animals, neuronal damage is mostly located in the cortex and hippocampus of the ipsilateral hemisphere, whereas in immature animals, significant neuronal loss is more widespread and includes ipsilateral hippocampus and ipsilateral thalamus, as well as the ipsilateral and the contralateral cortices compared to sham [72]. A noticeable hippocampal swelling was also observed 48 hours and 10 days after CCI in saline-treated animals. The decrease in the CCI-induced hippocampal swelling in animals post-treated with AL-8810 suggests involvement of the FP receptor in edema formation following brain trauma. One of the possible mechanisms is that overactivation of the FP receptor promotes the blood–brain barrier breakdown and aggravates edema formation [63,64]. Brain edema starts within minutes after TBI, and becomes progressively more severe over time, peaks at 24 hours after injury, and begins to decline after the third day [73,74]. Based on the increase in water content and Evans blue dye extravasation in the injured ipsilateral cortex and hippocampus, it has been suggested that the blood–brain barrier opening after CCI is biphasic [75]. The first blood–brain barrier breakdown occurs within four and six hours, and the second opening occurs on the third day after injury in the ipsilateral cortex and hippocampus [73]. However, the second opening of the blood–brain barrier does not contribute to a further increase in edema formation [73]. Our data indicating that repeated AL-8810 treatment provides no additional benefits compared with a single AL-8810 post-treatment is consistent with the putative role of only the first blood–brain barrier breakdown in edema development. Furthermore, these data suggest that the blockade of the FP receptor with a small-molecule antagonist at early time points might be an effective treatment for brain edema in TBI.

In addition, significant improvement in NDS and grip strengths were observed in the AL-8810-treated group compared with saline-treated animals. The improvements in NDS were dependent on AL-8810 dosage. The treatment was effective when the drug was administered following CCI at single dose, and additional AL-8810 injection did not cause significant improvement in NDS compared with single dosage. The grip strength test is included in the functional observational battery for use in the neurotoxicity studies in rodents to access neuromotor function [76]. In addition, clinical data indicate that grasping is impaired in children after TBI [77], and the quantitative analysis of precision-grip forces has been proposed as a sensitive method to assess recovery of fine motor skills [77,78]. Our data indicate a significant decrease in grip strength following CCI, and the decrease in grip strength was three-fold less in the AL-8810-treated group, which suggests that the FP receptor blockade might protect neuromotor function or facilitate its recovery following CCI.

Another important finding of this study is that anatomical brain injury is significantly reduced in FP^-/-^ mice. Interestingly, the anatomical improvement was not associated with the acute neurological outcome observed in AL-8810-treated WT mice. This finding suggests a dual role for the FP receptor in TBI recovery, and that activation of this receptor, at some level, might be beneficial for short-term outcomes. On the other hand, the beneficial effect of AL-8810 in WT mice could be explained by the unique pharmacological properties of this compound. In the studies performed in different cell lines, AL-8810, in addition to its antagonist properties, has been shown to be a partial agonist with approximately 20% efficacy at the endogenous FP receptor [23].

Although the FP receptors play important roles in different systems of mammalian organism overall, with a few exceptions, the effects of the FP receptor activation are believed to be pathophysiological; thus, more close attention may be paid to development of FP receptor antagonists as promising agents for treatment of diverse acute and chronic conditions, including preterm labor, cardiovascular disease, and fibroses (reviewed in [6]). Our data presented here indicate that a single treatment with AL-8810 applied immediately after CCI was sufficient to produce a beneficial effect on neurological outcome. Surprisingly, NDS outcomes after experimental TBI were not affected in FP^-/-^ mice compared to WT mice, indicating that the FP receptor may play a role in some aspects of neural behavior. This finding is in contrast with published data from our group obtained in stroke models where genetic deletion of the FP receptor improved neurobehavioral outcomes in models of ischemic stroke and excitotoxicity [21,22]. On the other hand, the published data with the prostaglandin E_2_ EP1 receptor, which has a similar structure and functions as the FP receptor [6,7,79], suggest that the effects of pharmacological blockade or genetic deletion of the EP1 receptor might be opposite in ischemic and hemorrhagic stroke [8,80-82]. Thereby, lack effect of FP knockout in TBI might be explained by the complexity of its pathology, which also includes a hemorrhagic component. Our data also suggest that resolving of acute hippocampal edema and gliosis, assessed by GFAP immunohistochemistry, lead to functional recovery and reduction of delayed inflammation and microglia activation in susceptible brain regions assessed by Iba1 immunohistochemistry. Thus, therapeutic strategy with a single-dose treatment would be optimal to obtain beneficial effects and minimize possible side effects in clinical applications.

In this study, significant improvements in the levels of microglial activation and reactive gliosis, assessed immunohistochemically, were detected in the selected brain regions of FP^-/-^ mice and WT mice that underwent treatment with AL-8810 following experimental TBI. In addition, in the FP^-/-^ mice, AL-8810 did not cause detectable changes in the Iba1 and GFAP immunoreactivities compared with saline (Additional file 1: Figure S1, B and C). Interestingly, similar beneficial effects of AL-8810 were observed with single and repeated treatment. Although further detailed studies are warranted to determine the exact therapeutic window of administration for the FP receptor antagonist in treatment of TBI, the data in the literature indicate that the upregulation of the inducible COX-2 enzyme occurs within several hours [12,14-16] and that the increased levels of the prostaglandin precursor remained for several days following brain trauma [18], suggesting a potential clinical application for prospective drugs in critical care. It is important to note that the role of COX-2 in brain injury is complex [15,16] and that the FP receptor antagonist selectively targets a downstream proinflammatory cascade of cyclooxygenase, leaving intact potentially neuroprotective ones. For example, we and others have previously shown that pathways involving prostaglandin PGE_2_ receptors EP2 and EP4 were demonstrated in preclinical excitotoxic and ischemic animal models [81,83,84].

## Conclusions

This study suggests that the PGF_2α_ FP receptor is involved in neuroinflammatory pathways and contributes to the overall pathology of brain trauma, and taking into account the similarity of prostaglandin drugs used in the clinic, selective FP antagonists could be used therapeutically in acute brain insult. Our findings provide a novel target for the treatment of TBI and help to elucidate the therapeutic potential and role of the FP receptor in acute brain trauma that will lead to more efficient and safe therapies; thus improving the quality of life of TBI patients for which effective treatment is currently unavailable.

## Abbreviations

ACA: Anterior cerebral artery; CCI: Controlled cortical impact; COX-2: Cyclooxygenase-2; CSF: Cerebrospinal fluid; FP-/-: FP receptor knockout; GFAP: Glial fibrillary acidic protein; Iba1: Ionized calcium-binding adapter protein 1; MCA: Middle cerebral artery; NDS: Neurological deficit scores; PaCO2: Partial pressure of carbon dioxide in arterial blood; PaO2: Partial pressure of oxygen in arterial blood; PBS: Phosphate-buffered saline; WT: Wildtype.

## Competing interests

The authors have no competing interests to declare.

## Authors’ contributions

AVG contributed to the study design, *in vivo* experiments, immunohistochemistry, data analysis, interpretation of results and writing of the manuscript; SWR contributed to the experiments and analysis of cerebrovascular morphology; CLB contributed to the immunohistochemistry, data analysis, and reviewing of the manuscript; SN contributed to the development of FP^-/-^ mouse; SD contributed to the study design, interpretation of results, and writing and revision of the manuscript. All authors have read and approved the manuscript for publication.

## Supplementary Material

Additional file 1: Figure S1 Lack of effects of AL-8810 post-treatment on brain pathology, gliosis, and microglia activation after sham surgery. **(A)** Representative photographs of bran sections from sham-injured WT mice post treated with a single dose of AL-8810 (10 mg/kg) 10 days after surgery stained with cresyl violet **(A)**, and DAB immunostained for GFAP **(B)** and Iba1 **(C)**. Top panels: examples of four brain sections cut throughout the entire craniotomy area. Middle panels: photographs of the stained and immunostained brain sections cut between 1 and 2 mm posterior from bregma zoomed in to demonstrate ipsilateral and contralateral susceptible brain regions (that is, cortical penumbra, hippocampus, and lateral dorsal and posterior nuclei of the thalamus). Bottom panels: zoomed area photographs presented in middle panels to demonstrate CA1 region of hippocampus, corpus callosum, and cortex in thinner details. Similar to saline-treated sham animals, no differences were observed between ipsilateral and contralateral sides in sham mice post-treated with AL-8810. The presented data is representative for a group of four mice. Click here for file
