# Supplementary material for: Prostaglandin F2α FP receptor antagonist improves outcomes after experimental traumatic brain injury
Source: J Neuroinflammation. 2013 Oct 30;10:132. doi: 10.1186/1742-2094-10-132 (PMC3907016; doi:10.1186/1742-2094-10-132)
Supplement: Additional file 1: Figure S1 — Lack of effects of AL-8810 post-treatment on brain pathology, gliosis, and microglia activation after sham surgery. (A) Representative photographs of bran sections from sham-injured WT mice post treated with a single dose of AL-8810 (10 mg/kg) 10 days after surgery stained with cresyl violet (A), and DAB immunostained for GFAP (B) and Iba1 (C). Top panels: examples of four brain sections cut throughout the entire craniotomy area. Middle panels: photographs of the stained and immunostained brain sections cut between 1 and 2 mm posterior from bregma zoomed in to demonstrate ipsilateral and contralateral susceptible brain regions (that is, cortical penumbra, hippocampus, and lateral dorsal and posterior nuclei of the thalamus). Bottom panels: zoomed area photographs presented in middle panels to demonstrate CA1 region of hippocampus, corpus callosum, and cortex in thinner details. Similar to saline-treated sham animals, no differences were observed between ipsilateral and contralateral sides in sham mice post-treated with AL-8810. The presented data is representative for a group of four mice. [file 1742-2094-10-132-S1.pdf]

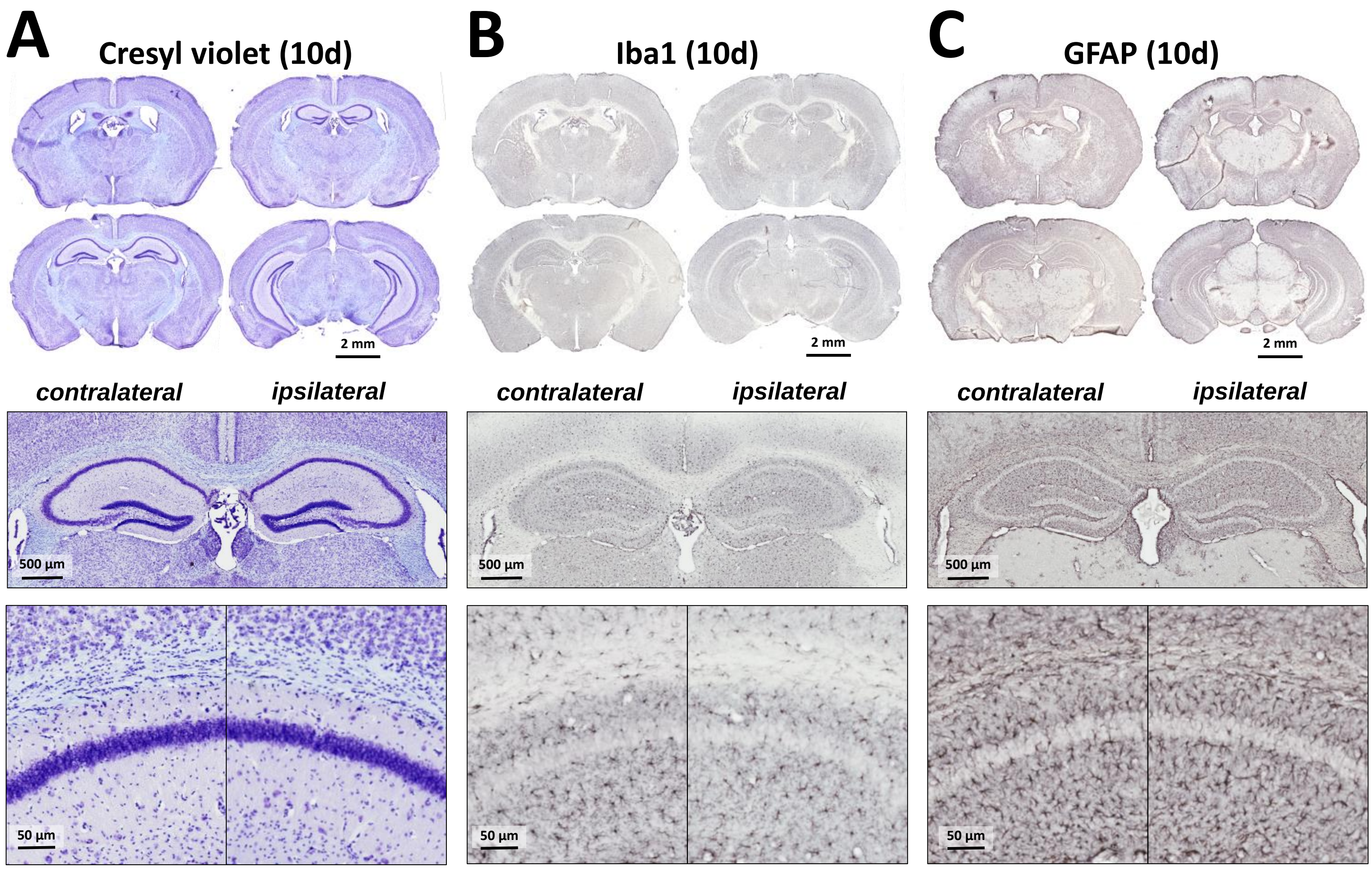

Additional file: Figure S1 Lack of effects of AL-8810 post treatment on brain pathology, gliosis, and microglia activation after sham surgery. (A) Representative photographs of bran sections from sham-injured WT mice post treated with a single dose of AL-8810 (10 mg/kg) 10 d after surgery stained with Cresyl violet **(A)**, and DAB immunostained for GFAP **(B)** and Iba1 **(C)**. Top panels: examples of four brain sections cut throughout the entire craniotomy area. Middle panels: photographs of the stained and immunostained brain sections cut between 1 and 2 mm posterior from bregma zoomed in to demonstrate ipsilateral and contralateral susceptible brain regions (i.e., cortical penumbra, hippocampus, and lateral dorsal and posterior nuclei of the thalamus). Bottom panels: zoomed area photographs presented in middle panels to demonstrate CA1 region of hippocampus, corpus callosum, and cortex in thinner details. Similar to saline-treated sham animals, no differences were observed between ipsilateral and contralateral sides in sham mice post treated with AL-8810. The presented data is representative for a group of four mice.
